# MicroRNA-214 in Health and Disease

**DOI:** 10.3390/cells10123274

**Published:** 2021-11-23

**Authors:** Meer M. J. Amin, Christopher J. Trevelyan, Neil A. Turner

**Affiliations:** Discovery and Translational Science Department, Leeds Institute of Cardiovascular and Metabolic Medicine, School of Medicine, University of Leeds, Leeds LS2 9JT, UK; meeramin64@gmail.com (M.M.J.A.); bs16cjt@leeds.ac.uk (C.J.T.)

**Keywords:** microRNAs, cardiovascular, cancer, bone, cell differentiation

## Abstract

MicroRNAs (miRNAs) are endogenously expressed, non-coding RNA molecules that mediate the post-transcriptional repression and degradation of mRNAs by targeting their 3′ untranslated region (3′-UTR). Thousands of miRNAs have been identified since their first discovery in 1993, and miR-214 was first reported to promote apoptosis in HeLa cells. Presently, miR-214 is implicated in an extensive range of conditions such as cardiovascular diseases, cancers, bone formation and cell differentiation. MiR-214 has shown pleiotropic roles in contributing to the progression of diseases such as gastric and lung cancers but may also confer cardioprotection against excessive fibrosis and oxidative damage. These contrasting functions are achieved through the diverse cast of miR-214 targets. Through silencing or overexpressing miR-214, the detrimental effects can be attenuated, and the beneficial effects promoted in order to improve health outcomes. Therefore, discovering novel miR-214 targets and understanding how miR-214 is dysregulated in human diseases may eventually lead to miRNA-based therapies. MiR-214 has also shown promise as a diagnostic biomarker in identifying breast cancer and coronary artery disease. This review provides an up-to-date discussion of miR-214 literature by describing relevant roles in health and disease, areas of disagreement, and the future direction of the field.

## 1. Introduction

Advances in genomic techniques, such as next-generation sequencing (NGS), have revealed that only 1–2% of the human genome encodes for proteins, as established by the ENCODE Project [1]. Despite this, 80% of the genome was deemed to have biological activity, which suggested that large regions had unknown functions not ascribed to protein coding. These elusive regions were largely referred to as non-coding RNAs (ncRNAs) and labelled as genetic ‘junk’ [2]. However, it has become increasingly apparent that ncRNAs perform major regulatory roles.

The most studied class of ncRNAs are the endogenously expressed, single-stranded microRNAs (miRNAs/miRs) which inhibit mRNA translation [3]. MiRNA processing involves cleavage by the Drosha and Dicer nucleases to form a miRNA:miRNA duplex, ultimately giving rise to ~22-nucleotide long miRNAs. These mature miRNAs are incorporated into the miRNA-induced silencing complex (miRISC), where miRNAs bind mostly to the 3′-untranslated regions (3′-UTRs) of mRNAs. This results in post-transcriptional suppression and reduces protein output. By manipulating the expression of multiple targets and gene networks, miRNAs can coordinate their effects to achieve a nuanced outcome, be it beneficial or detrimental. The combinatorial interactions between target genes and other miRNAs allows for a vast diversity of possibilities in modulating health and disease.

Lee and colleagues [4] discovered the first miRNA, called lin-4, in 1993, and it was found to regulate the temporal development of C. elegans. Many other miRNAs have since been identified, with ~2300 miRNAs in humans alone [5]. As of 2018, 38,589 pre-miRNAs across 271 organisms had been catalogued in miRbase; a number that is continuously rising [6]. Due to their relatively recent discovery and their vast numbers, little is known about each individual miRNA. Research has thus aimed to characterise their roles, but this is challenging as miRNAs have multiple targets and their functions can overlap with one another. Additionally, the potency of miRNA suppression can be undetectable in some circumstances [7]. Nonetheless, their importance in mammalian physiology remains uncontested, as inhibiting miRNA processing impairs early development and induces embryonic lethality in mice [8,9].

MiR-214 is encoded by an antisense ncRNA called Dynamin-3 opposite strand (Dnm3os) and was initially found to promote apoptosis in HeLa cells [10]. It has since been implicated in cardiovascular diseases, cancers, bone development and cell differentiation [11,12,13]. In the heart, miR-214 protects against Ca^2+^ ion overload [14] and oxidative damage [15], but downregulates angiogenesis [16], and potentially promotes pathological fibrosis [17,18]; although this is disputed [19,20,21]. MiR-214 variably affects cancer metastasis, proliferation and chemoresistance. It encourages gastric [22], ovarian [23], skin [24] and lung [25] cancer progression, but suppresses liver [16] and cervical [26] cancers. MiR-214 is also implicated in pancreatic cancer [27], myeloma [28] and nasopharyngeal carcinoma [29]. However, these lie outside the scope of this review, which instead focuses on cancers with abundant literature. MiR-214 also inhibits bone formation [30], and promotes the differentiation of skeletal [31] and vascular smooth muscle cells [32]. Not only does miR-214 act through a multitude of targets, but targets such as the tumor suppressor phosphatase and tensin homolog (PTEN) exhibit ubiquity in multiple conditions including oxidative stress [15], gastric and ovarian cancers [22,23], and T cell differentiation [33].

Unknowns surround the combinatorial interactions between miR-214 targets and other miRNAs. Additionally, large contradictions in the literature are yet to be settled, particularly in breast cancer [34,35], thus demanding more sensitive gain-of-function/loss-of-function studies to draw a definitive line. Transfecting cells with anti-miR-214 and pre-miR-214 (antagomir/agomir-214) allows for the cell-specific effects of miR-214 knockdown or upregulation to be observed, respectively [36]. Microarray analyses also highlight dysregulated miRNAs, often indicating disease interactions for further study.

This review aims to provide a broad overview of the literature surrounding the roles of miR-214 in health and disease, first by outlining miRNA biogenesis. Cardiovascular health, cancers, bone formation and cell differentiation are then discussed in the context of miR-214. Understanding these roles helps realise the potential in modulating miR-214 for therapeutic benefit. For instance, inhibiting miR-214 relieves cardiac hypertrophy [37], metastatic melanoma [38], and osteoporotic defects [30,39]. Given the devastation of these diseases globally, it is paramount to discover novel therapies. The pathogenic roles of miR-214 mean it can double as a therapeutic target and as an attractive diagnostic marker to identify severe diseases, such as breast cancer [40] and coronary artery disease (CAD) [41]. By bringing together information on all these diseases, we aim to provide a broad picture of the regulation and function of miR-214 across tissues, including molecular detail from specific diseases that may also be relevant to seemingly unrelated diseases.

## 2. MicroRNAs

### 2.1. Biogenesis and Action of MicroRNAs

**Figure 1:**
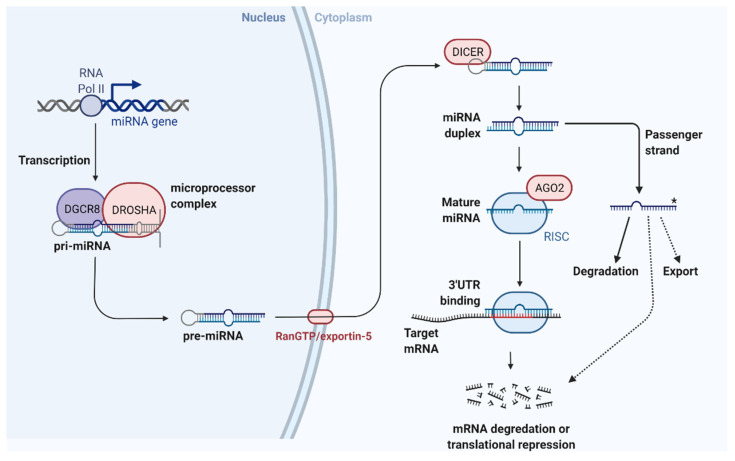
Classical miRNA biogenesis. RNA polymerases transcribe primary miRNAs (pri-miRNAs) which are then processed by the microprocessor complex minimally comprising RNase III Drosha nuclease with the RNA-binding DiGeorge Syndrome Critical Region 8 (DGCR8) cofactor. The resulting double-stranded precursor miRNA (pre-miRNA) is transported from the nucleus to the cytoplasm by RanGTP/exportin-5. A second RNase III nuclease called Dicer cleaves pre-miRNA at the stem-loop to form a miRNA:miRNA duplex. One strand from the duplex, known as the guide strand, is loaded into Ago proteins and the RNA-induced silencing complex (RISC). The other strand, called the passenger strand, is usually degraded, but in some cases may have functional activity within the cell, or be exported and act in a paracrine manner. The 5′-end of miRNA in the miRISC binds the 3′-UTR of target mRNAs by sequence complementarity. Messenger RNAs are then either translationally repressed or degraded. Adapted from ‘microRNA in Cancer’ by BioRender.com (2021). Retrieved from https://app.biorender.com/biorender-templates (accessed on 16 November 2021). For details, see [13,45].

Processing miRNAs is a multistep process, as illustrated in Figure 1. When transcribed into primary miRNAs (pri-miRNAs), the RNase III Drosha nuclease cleaves pri-miRNAs to form ~70-nucleotide pre-miRNAs [3]. Next, pre-miRNA is transported from the nucleus to the cytoplasm and is cleaved by the RNase III Dicer to form a duplex of ~22-nucleotide mature miRNAs [3]. Mature miRNAs are denoted with a -3p/-5p suffix, depending on their origin from the 3′/5′ end of the pre-miRNA hairpin, and an asterisk has traditionally been used to distinguish the less prevalent form [3]. One strand from the duplex is preferentially loaded into Argonaute (Ago) proteins and forms the miRISC. The balance of miRNA-3p/5p loading depends largely on cellular contexts. Most miRNAs are processed this way, however other pathways have been described. Introns can mimic pre-miRNA structures, known as ‘mirtrons’, to bypass Drosha/DGCR8 processing in flies and nematodes [42]. Alternatively, the miR-451 precursor is cleaved by Ago2 and matures independently of Dicer [43].

Through sequence complementarity, the 5′-end of miRNAs binds the 3′-UTR of target mRNAs for post-transcriptional suppression [3]. The degree of complementarity determines whether mRNAs are cleaved by Ago2 or targeted for degradation through miRISC-mediated decapping and deadenylation. Interestingly, the 3′-end of miRNAs can target 5′-UTRs, so miRNAs could potentially target both 3′- and 5′-UTRs [44].

### 2.2. Investigating MicroRNAs

High-throughput miRNA analysis is performed through microarrays, which profiles the expression of many miRNAs from tissue or fluid samples [45]. Quantitative real-time PCR (qRT-PCR) and Northern blotting quantifies specific miRNAs. Sensitive NGS and RNA-Seq can also identify novel miRNAs. Bioinformatic approaches using freely available databases (for example, miRBase.org and targetscan.org) can predict miRNA targets through analysing complementarity with 3′-UTRs. Predictions are further verified by Western blotting or luciferase assays, where the desired 3′-UTR is cloned into a luciferase reporter. A target is confirmed when luciferase expression is reduced upon introduction of the miRNA.

For functional analysis, miRNAs can be downregulated or upregulated by transfecting antagomirs/anti-miRs with antisense complementarity, or agomirs which are pre-miRNA mimics, respectively [36]. Genetic deletion in transgenic animals allows for studying systemic miRNA underexpression. However, cell-specific knockdown or modifying promoters to overexpress miRNAs encourages more selective in vivo examinations. Alternatively, miRNA ‘sponges’ such as the long non-coding RNAs (lncRNAs) MALAT1 [46] and X-inactive specific transcript (XIST) [47] can occupy miRNA binding sites to prevent target interactions, without affecting expression. Manipulating miRNA expression can come with unintended effects, and studies must build a comprehensive understanding of miRNAs without over-reliance on just one method.

### 2.3. MicroRNA-214

**Figure 2:**
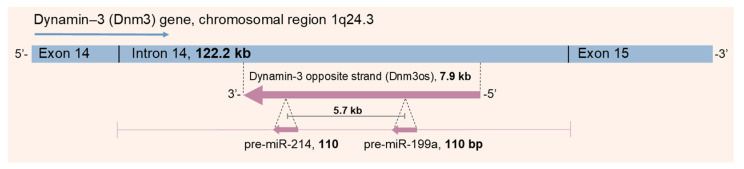
Illustration of the human *DNM3* gene and the miR-214/199a cluster. Schematic representation of the 7.9 kb long DNM3os transcript within intron 14 of the *DNM3* gene, encoded by the *DNM3* complementary strand. DNM3os co-expresses the clustered pre-miR-214 and pre-miR-199a, which are 5.7 kb apart. Adapted from [12,51].

Dnm3os is an antisense ncRNA conserved between vertebrates and was first identified within intron 14 of the *DNM3* gene in mice; human *DNM3* is in chromosomal region 1q24.3 [11,48] (illustrated in Figure 2). Mice with mutant Dnm3os exhibited developmental and skeletal defects, highlighting the essential role of Dnm3os [49]. Dnm3os encodes both pre-miR-199a and pre-miR-214, as evidenced by their downregulation in these Dnm3os mutants. Dnm3os is highly expressed during murine embryogenesis, and Loebel and colleagues [48] reported a downregulation of Dnm3os in embryos lacking the Twist transcription factor. This is corroborated by reduced miR-199a/miR-214 expression following Twist1 knockdown in ovarian cancer cells [50].

When co-transcribed, the miR-214/miR-199a cluster produces four mature miRNAs: the guide strands miR-199a-5p and miR-214-3p, and the passenger strands miR-199a-3p and miR-214-5p [11]. This review focuses on miR-214-3p (referred to as miR-214 throughout), however miR-214-5p (also termed miR-214*) is implicated in liver cancer [52], pancreatic cancer [53] and hepatic fibrosis [54]. More studies are required to understand how miR-214* interacts with miR-214. MiR-214 has now been characterised in cardiovascular diseases [11], cancers [12], osteogenesis [55] and myogenesis [56]. Table 1 summarises the discussed targets of miR-214 and their resulting effects.

## 3. MicroRNA-214 in Cardiovascular Physiology and Pathophysiology

**Figure 3:**
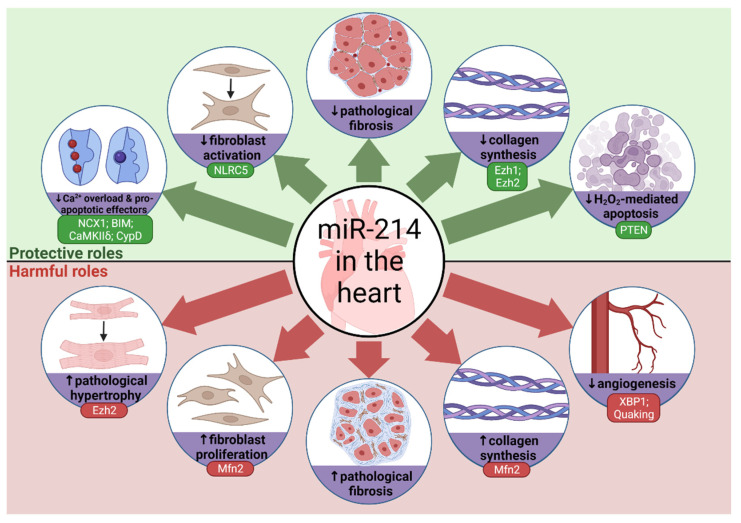
Effects of miR-214 on cardiovascular physiology. Summary of the protective and detrimental effects of miR-214 in the heart, with known target mRNA/proteins indicated, as discussed in the main text. Some effects only apply to specific cells or experimental conditions. See the main text of Section 3 for detailed information and references. Created with BioRender.com.

The diverse effects of miR-214 on cardiovascular health and disease are summarised in Figure 3 and discussed in more detail below.

### 3.1. Calcium Overload

**Figure 4:**
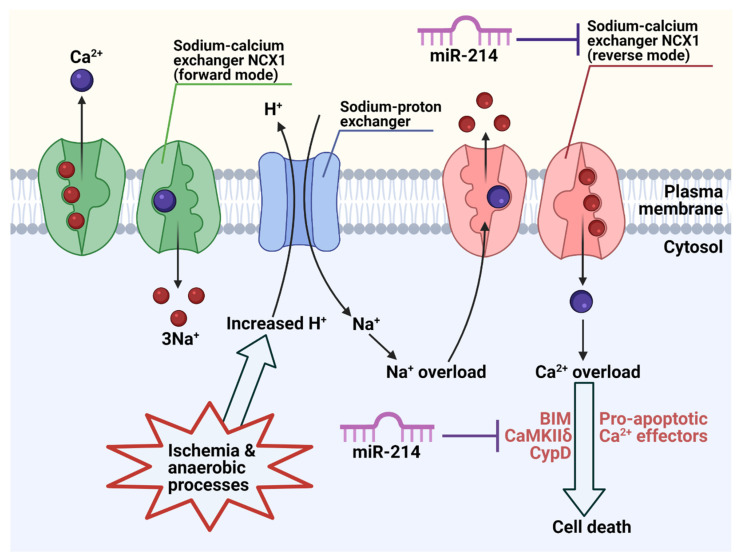
Calcium ion transport during ischaemic stress. The reversible sodium-calcium ion exchanger (NCX1) normally works in forward mode to take Ca^2+^ out of cells and bring 3Na^+^ into cells. During ischaemia, anaerobic processes produce an excess of intracellular protons (H^+^), which in turn stimulates sodium-proton exchange (Na^+^/H^+^) to remove H^+^ from the cell and bring Na^+^ in. The resulting influx of Na^+^ creates a Na^+^ overload which stimulates NCX1 to act in reverse. Consequently, 3Na^+^ are removed from the cell in exchange for bringing Ca^2+^ in. The overload of intracellular Ca^2+^ leads to cell death through the action of downstream proapoptotic effectors. Ca^2+^/calmodulin-dependent protein kinase II delta (CaMKIIδ) is involved in excitation–contraction coupling. Bcl-2-like protein 11 (BIM) promotes mitochondrial-dependent apoptosis which can result from the opening of the mitochondrial permeability transition pore (mPTP), which is where CypD acts as a regulatory protein. MicroRNA-214 can target and inhibit the 3′-untranslated region (3′-UTR) of NCX1, thus preventing reverse transport and attenuating Ca^2+^ overload. Additionally, microRNA-214 can directly target and inhibit BIM, CaMKIIδ and CypD, altogether diminishing apoptotic cell death. Created with BioRender.com. Information from [14,92,93,94].

Calcium ions (Ca^2+^) are well established in cardiac physiology, and rising intracellular Ca^2+^ is observed during ischemic/reperfusion (IR) injury [91]. Ischaemia results from inadequate blood supply to the cardiac tissue, and reperfusion causes damage upon reoxygenation. An overload of Ca^2+^ can cause heart failure and cardiomyocyte death through triggering mitochondrial-dependent apoptosis and autophagy during IR injury. The Na^+^/Ca^2+^ exchanger (NCX) maintains ion homeostasis, normally by transporting Ca^2+^ out of cardiomyocytes [92]. However, NCX can also reverse pump Ca^2+^ into cells. The direction of Ca^2+^ transport during ischaemia was heavily debated, but Tani and Neely [93] revealed links between Na^+^/Ca^2+^ and Na^+^/H^+^ exchange. Increased H^+^ (protons) from ischemic anaerobic processes forces the NCX to work in reverse to take excess Na^+^ out of the cardiomyocyte and bring Ca^2+^ in, as illustrated in Figure 4. Imahashi and colleagues reported that NCX knockout (KO) mice experienced less necrotic cell death, improved cardiac contractility and superior ATP metabolism [92]. This suggests the damaging nature of Ca^2+^ overload, where the absence of NCX may offer protection.

The role of miR-214 in IR injury was poorly defined until genetic loss-of-function studies showed that cardiac dysfunction and apoptosis were more apparent in miR-214 KO mice undergoing IR, indicating a protective function for miR-214 [14]. This appears contradictory to the findings of Cheng and colleagues who suggested that miR-214 promoted apoptosis in HeLa cells [10]. However, this is likely due to differences between in vitro and in vivo models, and the expression levels and importance of miR-214 in different cell types.

Vila-Petroff and colleagues [95] found that inhibiting reverse mode NCX prevents IR-mediated cardiomyocyte death. Supporting this, Aurora and colleagues [14] used luciferase assays to show that miR-214 targets the 3′-UTR of NCX1, and IR-induced apoptosis was increased in miR-214 KO cardiomyocytes. Additionally, increased intracellular Ca^2+^ in miR-214 KO cardiomyocytes was observed when stimulating the reverse mode of NCX1 through exposure to high extracellular Ca^2+^. This suggests that cardiomyocytes are vulnerable to Ca^2+^ overload and IR injury in the absence of miR-214, as NCX1 is no longer repressed. Contradicting this, Hampton and colleagues [96] found NCX1 to protect against IR stress, where transgenic mice overexpressing NCX1 had better preserved cardiac function. Both studies are not antithetical, as the former focuses on miR-214 absence, while the latter analyses NCX1 overexpression. Additionally, NCX1 transport is secondary to sarcoplasmic reticulum activity, so the full scope of Ca^2+^ homeostasis should be considered in future studies. Some studies favour NCX1 in withstanding cardiac stress [96,97], while others suggest NCX1 exacerbates Ca^2+^ overload and cardiac damage [14,92,93,95]. Discrepancies may be due to inconsistent cardiac stress conditions, and the organisms or cells tested.

Nonetheless, additional cardiac protection is demonstrated by miR-214 targeting downstream Ca^2+^ effectors like cyclophilin D (CypD), Bcl-2-like protein 11 (BIM) and Ca^2+^/calmodulin-dependent protein kinase II delta (CaMKIIδ) [14], as shown in Figure 4. In miR-214 KO mice, all three effectors had elevated expression. Furthermore, luciferase constructs confirmed that miR-214 targets the 3′-UTR of genes encoding CaMKIIδ and CypD, but surprisingly this was not seen with BIM. BIM was later identified as a direct target of miR-214 in nasopharyngeal carcinoma [94], although further verification would solidify these findings. CaMKIIδ regulates excitation–contraction coupling and inhibiting CaMKIIδ reduces the death of cardiomyocytes during IR injury, suggesting a detrimental role of CaMKIIδ in the heart [95]. BIM is a member of the Bcl-2 family which promotes mitochondrial-dependent apoptosis [14]. Furthermore, Ca^2+^ overload can open the mitochondrial permeability transition pore (mPTP), through which proapoptotic cytochrome c is released [98]. CypD is a regulatory protein in the mPTP, and CypD KO mice increases the difficulty for Ca^2+^ to open the mPTP, as reviewed in [99]. Overall, the absence of miR-214 which encourages reverse NCX1 transport, accumulation of intracellular Ca^2+^ and expression of proapoptotic effectors, suggests that miR-214 protects against IR injury and cardiomyocyte death. This presents potential therapeutic applications for cardiac damage using miR-214.

### 3.2. Reactive Oxygen Species

Cardiac damage during IR injury is further exacerbated through reactive oxygen species (ROS), which are primarily produced from complex I and III of the mitochondrial electron transport chain [100]. In the cardiovascular system, ROS plays a major pathological role. A detrimental cycle occurs when ischaemia damages the mitochondrial electron transport chain resulting in electron leakage, which helps generate free radicals from residual oxygen and then the newly generated ROS impairs the mitochondria even further [101]. Moreover, ROS can trigger apoptosis by opening the mPTP to release proapoptotic cytochrome c [98]. ROS generation can occur in cardiomyocytes at the onset of ischaemia, as well as with reperfusion [102]. 

Regarding miR-214, one study revealed that it confers cardioprotection against ROS-mediated damage, specifically hydrogen peroxide (H_2_O_2_) [15]. Previously, associations between miR-214 and H_2_O_2_ were poorly defined. However, miR-21 had exhibited protection against H_2_O_2_, suggesting the involvement of other miRNAs [103]. H_2_O_2_ increased apoptosis rates in rat cardiomyocytes and miR-214 levels were elevated in response to H_2_O_2_ exposure, signifying a protective role for miR-214 [15]. Flow cytometry revealed that transfecting cardiomyocytes with anti-miR-214 increased H_2_O_2_-mediated apoptosis. Additionally, PTEN levels and apoptosis decreased with pre-miR-214 transfection. These findings support the conclusion that miR-214 is stimulated upon H_2_O_2_ exposure, and targets PTEN to protect cardiomyocytes from H_2_O_2_-mediated apoptosis. However, no luciferase assays were carried out, thus leaving it to other studies to confirm that miR-214 directly targets PTEN [22]. Interestingly, miR-214 could potentially exacerbate oxidative stress in the aging murine liver, as detoxifying glutathione S-transferase levels negatively correlated with miR-214 [104]. However, functional evidence of miR-214 is required in addition to investigating other aging organs, such as the heart.

PTEN was first identified in 1997, where it was characterised as a tumour-suppressor gene located on the chromosome region 10q23 [105]. Shortly after, it was reported that PTEN suppressed cell migration and spreading [106]. PTEN may also promote caspase-3 expression to induce tumour cell apoptosis in gastric cancer [107]. Furthermore, decreased PTEN expression was associated with elevated cell motility and tumourigenic angiogenesis. Therefore, miR-214-targeting of PTEN is clinically relevant in cancer progression and presents therapeutic possibilities for ROS-mediated cardiac damage.

### 3.3. Hypertrophy and Angiogenesis

MiR-214 in IR injury has been well described, but uncertainty surrounds miR-214 in non-ischaemic cardiac damage. Multiple miRNAs, including miR-214 were upregulated in hypertrophic mouse hearts, suggesting links between miRNAs and hypertrophy [18,108,109]. Hypertrophy leads to enlarged cardiac tissue, often in response to stress, e.g., myocardial infarction, hypertension or physical exercise [110]. Protein synthesis is increased, and growth factors are secreted to bolster cardiac contractility. Increased myocardial mass demands greater oxygenation, and angiogenic signalling helps meet these demands by forming new blood vessels from pre-existing structures. Angiogenesis is regulated by factors such as vascular endothelial growth factor (VEGF) and placental growth factor (PLGF). Without proper angiogenesis in a hypertrophic heart, hypoxia occurs due to inadequate vascular oxygen delivery, eventually resulting in heart failure. 

Significant miR-214 upregulation was reported in human heart failure patients and in hypertrophic mice hearts [16], which agrees with previous studies [108,109]. This suggests a pathological role of miR-214 in hypertrophy. Luciferase assays confirmed that miR-214 targeted the 3′-UTR of Ezh2, a repressor of cardiac hypertrophy [37]. Here, antagomir-214 rescued mice from pathological hypertrophy, therefore highlighting the therapeutic benefits of miR-214 inhibition in pressure-overloaded hearts. 

During stimulated hypertrophy, miR-214 inhibition increased vascular density [16]. X-box binding protein (XBP1) was found to be targeted by miR-214 here, as evidenced by luciferase assays in human umbilical vein endothelial cells (HUVECs). XBP1 is a transcription factor that regulates cell growth and resolves ER stress via the unfolded protein response. Overexpressing XBP1 upregulated tube formation in HUVECs, and transfection of XBP1 rescued cells from anti-angiogenic miR-214. XBP1 is also involved in inflammation and energy metabolism, thus prompting study into other effects of miR-214. This experiment is limited to HUVECs, so miR-214 in different cell types should be examined in the future to support these findings. Nonetheless, there is agreement with other studies. The pro-angiogenic factor Quaking is targeted by miR-214, and silencing miR-214 increases angiogenic cell sprouting during murine retinal development [77]. MiR-214 also inhibits angiogenesis in human hepatocellular carcinoma (HCC) [67]. Pro-angiogenic ginsenoside-Rg1 is a component of the Chinese medicine ginseng, and it downregulated miR-214 and upregulated the angiogenic regulator endothelial nitric oxide synthase (eNOS) in HUVECs [111]. Further studies should investigate whether miR-214 directly antagonises eNOS. 

Finally, Lu and colleagues [41] discovered that circulating miR-214 was reduced in patients with CAD. Therefore, miR-214 could act as a diagnostic biomarker for identifying CAD. Moreover, the expression of pro-angiogenic PLGF was increased, indicating another potential miR-214 target. However, cautious interpretation is recommended, firstly because of the small sample size. Secondly, circulating miR-214 can fluctuate with differential expression and uptake by cells. Future studies should examine circulating miR-214 levels, ideally with a larger cohort of CAD patients. Altogether, abundant research evidences the anti-angiogenic properties of miR-214. Downregulating cardiac angiogenesis leads to hypertrophic hearts being unable to maintain function under stress, ultimately leading to heart failure. These studies support the development of therapies based on blocking miR-214 to reverse insufficient angiogenesis. Targeting miR-214 has been tested in vivo, where adeno-associated virus serotype 9 (AAV9)-mediated delivery of anti-miR-214 restored cardiac function in hypertrophic mouse hearts [16]. Conversely, upregulating miR-214 could be of value to attenuate harmful tumour angiogenesis. More work is needed on in vivo models and cultured human cells before taking miR-214 to clinical settings, however.

### 3.4. Fibrosis

Cardiac reparative fibrosis is a normal process of remodelling following damage such as IR injury or acute myocardial infarction (AMI) [112]. Fibrosis is primarily mediated by cardiac fibroblasts and the deposition of extracellular matrix (ECM) components in the myocardium [112,113]. The ECM is a complex network critical for connecting cells, strengthening cardiac structure, dissipating mechanical force, and distributing electrical signals. The deposition of fibrillar collagen, a major ECM component, replaces dead cardiomyocytes and allows myocardial remodelling to restore contractility following damage. Increased type I and type III procollagen mRNA was observed in an early study on infarcted rat hearts [114]. However, fibrosis can be detrimental when the synthesis and degradation of the ECM components is imbalanced [115]. Reactive fibrosis in a pressure-overloaded heart results in an overproduction of growth factors to support myocytes in response to an increased workload. This can cause stiffened ventricles and dysfunctional diastolic relaxations and systolic contractions. Conversely, collagen breakdown by matrix metalloproteinases (MMPs) likely accelerates the progression of left ventricular hypertrophy to heart failure [116]. 

The primary effector cells of cardiac fibrosis are fibroblasts, which are well established in synthesising and degrading ECM components [112,113]. Fibroblasts can differentiate into mobile myofibroblasts that contract collagen and express alpha-smooth muscle actin (α-SMA) to facilitate wound closure. Fibroblasts can secrete cytokines and growth factors such as interleukin-1β (IL-1β), IL-6, and tumour necrosis factor-α (TNF-α) [117,118]. Introducing IL-1β and TNF-α to rat cardiac fibroblasts decreased collagen synthesis and increased MMP expression, overall, promoting collagen turnover [119]. Similarly, increased ECM turnover was seen when exposing fibroblasts to oxidative stress such as H_2_O_2_ [120]. Understanding the regulation of ECM components may highlight novel drug targets. 

Hou and colleagues [121] observed miRNA expression being mediated by β-adrenergic receptor (β-AR). Isoproterenol (ISO) is a β-AR agonist, whereas propranolol antagonises β-AR. Hearts from ISO-treated rats mostly had upregulated miRNAs, including miR-214, and downregulation was prevalent in the propranolol group. This suggests that miRNAs are promoted by β-AR signalling. Additionally, the inhibition of β-AR signalling by HIP-55 impaired the ERK1/2-MAPK pathway and cardiac fibroblast proliferation during ISO treatment [122]. Moreover, overexpressing β-AR in transgenic mice resulted in cardiac hypertrophy and large fibrotic regions [123]. 

In agreement with Hou and colleagues [121], miR-214 was upregulated upon ISO treatment in mice [17]. Luciferase assays from this study showed that miR-214 targeted mitofusin2 (Mfn2), an inhibitor of the proliferative ERK1/2-MAPK pathway [17]. MiR-214 knockdown via antagomir-214 significantly decreased ISO-induced cardiac fibroblast proliferation and collagen synthesis, and the opposite was shown with agomir-214. This suggests that miR-214 could promote pathological fibrosis through enhanced fibroblast proliferation and collagen deposition. Additionally, in our own study, fibroblast-specific KO of p38α MAPK in mice attenuated the ISO-mediated upregulation of miR-214 and detrimental fibrotic remodelling, suggesting a pathological role for miR-214 in fibrosis [18]. Similarly, Denby and colleagues [124] found that antagonising miR-214 through genetic deletions and antagomirs diminishes renal fibrosis. Intriguingly, miR-214* is also associated with liver fibrosis [54]. Overexpressing miR-214* upregulated MMPs and α-SMA in human liver cells, thus prompting further investigation of miR-214*.

Despite these findings, recent studies have reported that miR-214 confers cardioprotection against fibrosis. In vivo, the fibroblast-specific genetic deletion of miR-214 resulted in greater collagen deposition and fibrosis when mice were subjected to transverse aortic constriction (TAC); a technique to simulate cardiac hypertrophy [21]. The same study showed that miR-214 inhibited fibroblast activation in vitro by targeting the NOD-like receptor family CARD domain containing 5 (NLRC5). Upon fibrosis-inducing angiotensin-II treatment, the transfection of pre-miR-214 into cardiac fibroblasts led to reduced collagen I/III and pro-fibrotic transforming growth factor-β1 (TGF-β1) expression [20]. Similar observations were also seen with in vivo AMI models. This was supported by Zhu and colleagues, who reported a reduction in cardiac fibrosis with agomiR-214 in angiotensin-II treated mice [19]. In addition, miR-214 inhibited collagen type I alpha 1 and 3 (Col1a1, Col1a3) expression in myofibroblasts by targeting Ezh1 and Ezh2; the enzymatic components of the transcription-suppressing PcG proteins [19]. Together, these studies, through in vitro and in vivo models, have shown that miR-214 attenuates cardiac fibrosis. This is a strong hypothesis, and it suggests a therapeutic benefit in promoting miR-214 for fibrotic protection, however the findings of Sun and colleagues [17] remain contradictory. A likely explanation for this may be the unique stimuli in different cardiac models and tissue-specific phenotypes. The neurohumoral stimulation of β-AR by ISO differs from TAC which mimics a pressure-overloaded heart, and this differs from AMI models which simulate ischaemia/reperfusion. AMI is potentially more likely to induce reparative fibrosis, whereas ISO/TAC models encourage reactive fibrosis. Future studies should clarify these disagreements with further in vivo models to replicate multiple fibrotic environments. The discrepancies between miR-214 knockdown by antagomirs or genetic deletions should also be explored.

## 4. MicroRNA-214 in Cancer Progression

**Figure 5:**
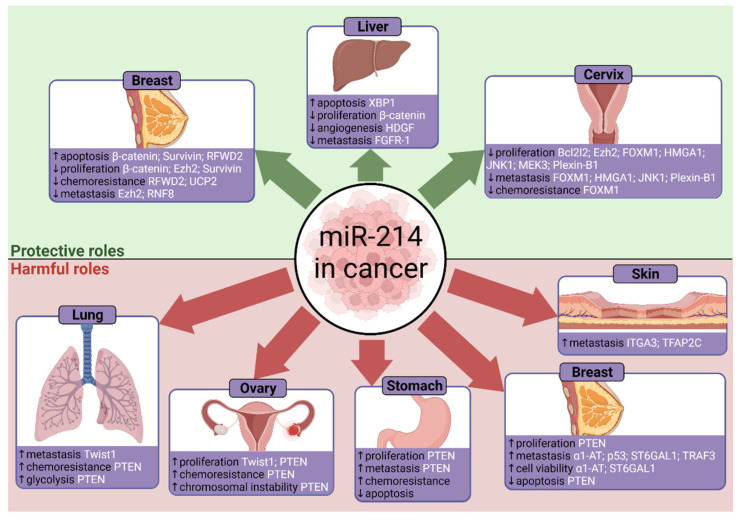
Effects of miR-214 on cancer progression. Summary of the protective and detrimental effects of miR-214 on cancer progression, with known target mRNA/proteins indicated, as discussed in the main text. Note that Twist1 is an exception as it is not targeted by miR-214 but instead modulates and upregulates miR-214 to achieve the corresponding effects. Some effects only apply to specific cells or experimental conditions. See the main text of Section 4 for detailed information and references. Created with BioRender.com.

The various effects of miR-214 on cancer progression are summarised in Figure 5 and discussed below.

### 4.1. Gastric Cancer

According to 2018 GLOBOCAN estimates, gastric cancer caused 783,000 deaths worldwide [125]. It is the third leading cause of cancer deaths behind colorectal and lung cancers. Aside from nutrition and alcohol intake, the bacterium Helicobacter pylori remains the leading risk factor for gastric cancer. Infection with H. pylori causes gastritis and inflames the stomach lining and epithelial cells; this causes ulcers and eventually leads to gastric adenocarcinomas. Interestingly, eradicating H. pylori can prove detrimental. Gastric cancers are classified into cardia and non-cardia subtypes. Over 90% of non-cardia cases are associated with H. pylori, however the bacterium has shown protection against cardia-type cancers by reducing acid secretion and oesophageal gastritis. Hence, eradication of H. pylori reduces non-cardia incidence but increases the prevalence of cardia cancers [125]. Emphasising diet and lifestyle modifications is likely a better alternative which has seen noticeable reductions in gastric cancer mortality [126].

Analysing microarrays from 353 human gastric cancer samples found that high miR-214 expression correlated with a low patient survival [127]. This suggests that miR-214 modulates cancer, however the underlying mechanisms were unknown at the time. Additionally, this study was limited by only taking samples from Japan, and the patient demographic could lead to genetic bias in miRNA expression profiles. Nevertheless, this prompted further study of miR-214 in gastric cancer. 

Supporting the findings of Lv and colleagues [15], who found that the tumour suppressor PTEN was downregulated upon miR-214 overexpression, Yang and colleagues reported similar observations from RT-PCR of human gastric cancer tissues [22]. Additionally, miR-214 correlated with poor clinical outcomes and metastatic gastric cancer, which is consistent with other reports [127]. Luciferase assays confirmed that miR-214 targets PTEN, and silencing PTEN by RNA interference attenuated the anti-miR-214-mediated inhibition of proliferation and migration [22]. This suggests miR-214 downregulates PTEN signalling to hasten gastric cancer progression. These findings present therapeutic interventions without eradicating H. pylori, as suppressing miR-214 could impair proliferation and metastasis. More recently, Xin and colleagues demonstrated strong correlations between miR-214 downregulating PTEN with increased peritoneal metastasis in human gastric cancer cells [73]. A separate study associated PTEN with promoting apoptosis through capase-3 in gastric cancer, suggesting that miR-214-mediated targeting of PTEN could inhibit antitumourigenic apoptosis [107]. Finally, miRNAs often have multiple targets, so outcomes are multifactorial. In zebrafish, it was discovered that miR-214 inhibited the suppressor of fused, or Su(fu) [84]. This activates hedgehog signalling which is involved in regulating cell differentiation and gastric cancer progression, as reviewed in [128]. 

Another significant aspect of gastric cancer mortality is chemotherapy resistance (chemoresistance) [129]. This includes resistance to cisplatin (DDP) which impairs DNA replication in cancerous cells. Resistance is brought about by uptake/efflux transporters, functional changes in drug targets, enhanced DNA repair, and evasion of apoptosis. Remarkably, DDP resistance in gastric cancer can be reversed by delivering anti-miR-214 in membrane-derived vesicles (exosomes), resulting in reduced tumour sizes and increased apoptosis in mice [130]. Exosomes bypass the cytotoxicity and poor delivery seen in conventional viral or liposomal vectors. Despite this, the molecular mechanisms for miR-214-mediated chemoresistance in gastric cancer are not well known, thus demanding additional study. Eventually, this may enhance patient response to chemotherapy and reduce mortality rates.

### 4.2. Liver Cancer

Hepatocellular carcinoma (HCC) is the most common primary liver cancer [125]. Globally, liver cancer was estimated to cause 782,000 deaths in 2018, making it the fourth deadliest cancer. Risk factors include hepatitis type B or C viruses (HBV, HCV), and lifestyle choices like smoking and diet.

In HCC cell lines, miR-214 was downregulated in 65% of cases [89], with similar observations reported in HCC samples [67]. XBP1, a regulator of the UPR, was again identified as a miR-214 target in HCC cell lines [89]. The UPR responds to ER stress resulting from the damaging accumulation of misfolded proteins, and XBP1 is important for tumour survival under stressful conditions like hypoxia. IRE1 splices an intron from XBP1 pre-mRNA to activate XBP1. In mice, XBP1 knocks out severely impaired tumour growth during hypoxia [131]. Similarly, inhibiting IRE1 in myeloma cells diminishes active XBP1, thus increasing their sensitivity to apoptosis during ER stress [132]. Future studies should investigate other UPR pathways like PKR-like ER kinase (PERK) or ATF6.

Nuclear factor-κB (NFκB) is a critical transcription factor that induces expression of cytokines and proinflammatory factors, and is involved in proliferation, apoptosis, inflammation, and immunity [133]. Normally, NFκB remains inactive in the cytoplasm by being bound to inhibitory IκB protein, thus preventing aberrant activation. IκB is phosphorylated when stimulated by factors like lipopolysaccharide (LPS) or viral components, thereby releasing inhibition of NFκB so it can migrate to the nucleus to bind promoters and upregulate gene expression. In hypoxia, greater NFκB activation correlated with increased IκB phosphorylation, suggesting cells respond to stress via NFκB signalling [134]. Additionally, NFκB may confer protection in HCC cell lines undergoing oxidative stress with H_2_O_2_ [133]. 

In HCC cell lines, miR-214 reduced proliferation and increased apoptosis [89]. Similar observations were found in vivo with agomir-214-treated mice. In this study, inducing the UPR or stimulating NFκB with LPS resulted in reduced miR-214. Conversely, inhibiting NFκB attenuated the downregulatory effects of the UPR on miR-214. Together, this suggests that the UPR downregulates miR-214 by activating NFκB. This prevents miR-214-mediated tumour suppression and XBP1 downregulation, thus encouraging HCC progression. This is consistent with Romero-Ramirez and colleagues who demonstrated the importance of XBP1 in tumour survival [131]. In cardiac myofibroblasts, NFκB signalling was instead found to upregulate miR-214 upon angiotensin-II treatment [19]. However, this contradiction is likely due to differing cell types. Interestingly, miR-214* can also suppress HCC metastasis, thus revealing another therapeutic possibility [52].

An abundance of studies has revealed other miR-214 targets, such as β-catenin [59]. Overexpressing miR-214 inhibits HCC growth, as confirmed in vivo from mice exhibiting smaller tumours. Notably, the expression of β-catenin and its downstream effectors were suppressed, including c-myc, TCF-1 and cyclin D1. In 43% of in vivo HCC samples, β-catenin accumulated in the nucleus where it activates the transcription of pro-proliferative genes like c-myc and cyclin D1 [135]. Investigating oncogenic β-catenin mutations with larger sample sizes can help understand HCC progression. Another miR-214 target is hepatoma-derived growth factor (HDGF), which promotes hypervascularity [67]. Here, miR-214 suppressed angiogenic tube formation in vivo and in vitro. Finally, fibroblast growth factor receptor 1 (FGFR-1) was downregulated and targeted by miR-214 in HCC, with cell invasion being reduced by >50% [64]. The diversity of targets in HCC suggest that others exist for future studies to identify, alongside potential combinatorial interactions between targets. Taken together, miR-214 acts to suppress HCC progression as it inhibits proliferation, tumour growth, angiogenesis, and metastasis through targeting XBP1, β-catenin, HDGF and FGFR-1. Overexpressing miR-214 is therefore an attractive strategy for countering HCC.

### 4.3. Ovarian Cancer

Ovarian cancer was estimated to cause over 180,000 deaths in 2018 [125], with a greater proportion of deaths:cases than cervix or uterine cancers. Risk factors include age, menstruation cycles, endometriosis, diet and obesity, among others. Epithelial ovarian cancer (EOC) is the most common subtype, with the tumour originating from epithelial cells [136]. EOC cell populations are characterised as chemoresistant, self-renewing type I cells or matured type II cells [50]. Stem cell-like type I cells can differentiate into type II cells, and understanding this transition is important to tackle recurrent tumours.

In human ovarian tumours, miR-214 was one of the most commonly upregulated miRNAs [23]. PTEN was found to be targeted by miR-214 here, and downregulating PTEN increases phosphorylation of the prosurvival Akt pathway. Consequently, miR-214 promoted cisplatin chemoresistance, thereby encouraging tumour recurrence. However, a larger sample size of ovarian tumours would help provide more evidence. MiR-214 also targets and downregulates the ubiquitin ligase RNF8 to impair the DNA damage response and promote tumourigenic chromosomal instability in ovarian cancer cells [81].

Twist1 is a transcription factor that regulates cell differentiation, inflammation, and apoptosis [51]. Importantly, Twist1 was shown to regulate the expression of the miR-199a/miR-214 cluster during embryonic mice development. Furthermore, several studies have observed the parallel expression of miR-214 and miR-199a in ovarian cancer, where they are both increased [23] or both decreased [137], indicating that these miRNAs are clustered.

Expression of both miR-199a and miR-214 was elevated in type II EOC cells but decreased in type I cells [50]. Moreover, Twist1 expression was higher in type II cells. Knockdown of Twist1 correlated with decreased miR-199a/miR-214 expression, suggesting that Twist1 upregulates miRNA expression. Interestingly, Twist1 can negatively modulate NFκB signalling by upregulating miR-199a, which targets IKKβ. Later research identified that miR-214 is negatively regulated by the NFκB pathway in HCC, so a similar mechanism may exist in ovarian cancer [89]. Additionally, greater levels of phosphorylated-Akt and decreased PTEN were seen in mature type II cells, where miR-214 is more prevalent [50]. Overall, Twist1 is a complex modulator of the miR-199a/214 cluster, where it downregulates inflammatory NFκB signalling and upregulates pro-proliferative Akt signalling. Remarkably, inhibiting Twist1 can restore stem-cell-like properties in mature type II cells. Indeed, the in vitro differentiation of type I to type II cells correlated with increased Twist1, thus accelerating EOC progression by promoting proliferative cell phenotypes. Increased Akt signalling by miR-214 targeting PTEN was also confirmed by another group [23]. Manipulating miR-199a/miR-214 regulation and preventing Twist1 activity could ultimately slow down EOC development.

### 4.4. Cervical Cancer

Cervical cancer is the fourth leading cause of cancer death in women with over 300,000 global deaths in 2018 [125]. It is more common in less developed countries, partially due to poor screening and vaccination programmes. Vaccinations counter the high-risk human papillomavirus (HPV) subtypes, like HPV-16 and HPV-18. These viruses promote genetic instability and the degradation of tumour suppressors like p53 through their E6 and E7 proteins [138]. Ultimately, HPV facilitates cervical cancer progression and mortality.

Several miRNAs were reported to be dysregulated in human cervical cancer cells [139]. Furthermore, miR-143 and miR-145 suppressed proliferation in HeLa cells, whereas miR-146a promoted growth. It is plausible that miR-214 plays a role in cervical cancer, especially as it is dysregulated in ovarian cancer [23]. Supplementing the findings of Wang and colleagues [139], another study observed miR-214 downregulation in vivo and in vitro with human cervical cancer tissues and HeLa cells [26]. Similarly, low miR-214 expression was reported in HeLa cells containing HPV-16 DNA [138]. However, it should be noted that HeLa cells are not directly comparable to tissue samples, as these cells have been cultured over a long period of time and have altered genetically.

MiR-214 mediates the antiproliferative effects by targeting MEK3 and c-Jun N-terminal kinase 1 (JNK1) mRNAs, as verified by luciferase assays [26]. A recent study showed that the E6 oncogene of HPV activates JNK1 signalling to promote cell proliferation and metastasis in cervical cancer, partially from JNK1 upregulating epidermal growth factor receptor (EGFR) signalling [140]. Meanwhile, MEK3 activates the p38 MAPK pathway to promote apoptosis [141]. Cervical tumour suppression can therefore occur from miR-214 downregulating JNK1. However, miR-214 targets proapoptotic MEK3, which is unexpected if miR-214 supposedly mediates antiproliferation. Future studies should elucidate this interaction. Further confusion arises from contrasting findings of miR-214 underexpression in cervical cancers [26], but overexpression in cervical invasive squamous cell carcinomas (ISCCs) [142]. However, this could be due to tissue-specific physiological differences. The effect of high-risk HPV subtypes on miR-214 in cervical cancer requires further study, as HPV-16 can downregulate the potentially protective miR-218 [138]. Several other studies have reported miR-214 to suppress cervical tumour growth and metastasis through targeting and downregulating high mobility group AT-hook 1 (HMGA1) [68], Plexin-B1 [72], Bcl2l2 [62], Ezh2 [63], and forkhead box protein M1 (FOXM1) [66]. The tumour suppressive activities through a vast diversity of targets solidifies miR-214 to be an attractive therapy for cervical cancers.

### 4.5. Skin Cancer

An analysis of malignant melanoma trends, the most lethal skin cancer, has found that mortality has continuously increased over a 30-year period from 1985 [143]. Therefore, new interventions for melanoma treatment are necessary. Exposure to DNA-damaging ultraviolet radiation remains the biggest contributor to melanoma by transforming melanocytes to proliferating malignant cells [144]. Ultraviolet radiation can induce genetic mutations, impair tumour suppressors like p53, or dysregulate pathways such as MAPK. Immune checkpoint inhibitors and kinase inhibitors like trametinib are the primary means of melanoma therapy, however modulating miRNAs may prove clinically relevant.

An early experiment demonstrated differential miRNA expression in melanoma samples [145]. However, these were not compared to healthy samples, so these findings are difficult to interpret. Later, Mueller and colleagues [146] analysed microarrays from melanoma cell lines and found 49 strongly upregulated miRNAs during early melanoma progression. This indicates an association between miRNAs and the transformation to malignancy.

MiR-214 was highly expressed in melanoma cells [24], which is in agreement with previous findings [147,148]. In this study, transfection with pre-miR-214 increased cell invasion in vitro [24]. Furthermore, in vivo injection of overexpressing-miR-214 melanoma cells into mice tails increased lung metastases. Luciferase and expression assays in human melanoma samples confirmed that miR-214 targets the adhesion molecule integrin α3 (ITGA3) and transcription factor AP-2 gamma (TFAP2C). However, these findings were limited by the human data set only presenting mRNA, so verification of protein expression is required. Silencing TFAP2C or ITGA3 both increased in vitro cell migration, mirroring the action of miR-214. The protumourigenic effects of miR-214 are likely primarily through downregulating TFAP2C, as it regulates many factors. For example, TFAP2C represses pro-growth VEGFA but activates ERBB2 [24]. Unexpectedly, ITGA3 promotes tumour growth in breast cancer, yet the oncogenic miR-214 downregulates it in melanoma [149]. Further study of ITGA3 is needed, but discrepancies may be attributed to unique cellular contexts and tumour microenvironments. Focusing on human cell lines and in vivo tumour samples will help guide melanoma therapy. Moreover, the many factors regulated by TFAP2C require their effects on carcinogenesis to be clarified. For instance, miR-214 upregulates the activated leukocyte cell adhesion molecule (ALCAM) through negatively modulating TFAP2 and miR-148b [150]. Consequently, ALCAM promoted malignant melanoma progression. Orso and colleagues found that inhibiting miR-214 or overexpressing miR-148b can impair melanoma metastasis, due to downregulated ALCAM and ITGA5 adhesion molecules [38]; both of which are direct targets of miR-148b. Furthermore, simultaneously downregulating miR-214 and upregulating miR-148b blocked metastasis in melanoma and breast cancer cells, which presents a promising combinatorial therapy. Overall, the research supports miR-214 to promote melanoma progression and metastasis.

### 4.6. Lung Cancer

Worldwide, lung cancer causes the most cancer deaths with an estimated 1.7 million deaths in 2018, or 18.4% of all cancer deaths [125]. Aside from lifestyle changes and tobacco regulation, knowledge of lung carcinogenesis mechanisms can give insight for novel therapies. Current treatments include EGFR-tyrosine kinase inhibitors (EGFR-TKI), such as gefitinib or erlotinib [151,152]. Randomised phase 3 clinical trials have demonstrated that these EGFR-TKIs significantly prolonged survival in patients with activating EGFR mutations, and this was superior to standard chemotherapy protocols. Unfortunately, patients can acquire resistance to EGFR-TKIs. 

Microarray comparisons of primary lung cancer samples with healthy tissues revealed 43 differentially expressed miRNAs, with miR-214 being significantly upregulated (*p* = 8.6 × 10^−6^) [153]. MiR-214 was also upregulated in non-small cell lung cancer (NSCLC), which accounts for over 80% of lung cancer cases [74]. MiR-214 has been implicated in promoting cisplatin resistance in ovarian cancer [23] or reversing resistance in gastric cancer [130]. Additionally, miR-214 can indirectly affect EGFR signalling by targeting JNK1 [26]. Therefore, modulation of gefitinib resistance by miR-214 is plausible.

MiR-214 expression was significantly upregulated in the gefitinib-resistant lung cancer cell line HCC827, from observations of RT-PCR and Northern blotting [25]. Here, knockdown of miR-214 increased PTEN expression and decreased phosphorylation of the protumourigenic Akt pathway. Furthermore, miR-214 knockdown saw previously resistant EGFR-mutant lung cancer cells now sensitive to gefitinib, as shown in MTS viability assays. MiR-214 was confirmed to target PTEN, which supports similar conclusions from other studies [15,22,23,73]. Additional effects of miR-214 targeting PTEN include promoting glycolysis in NSCLC to generate energy for proliferating cancer cells [74]. As miR-214 may confer gefitinib resistance in lung cancer cells, downregulating miR-214 could counter EGFR-TKI resistance [25]. More recently, antagomir-214 was found to reverse NSCLC gefitinib resistance in vitro and in vivo [154]. Further in vivo studies of human cancer samples could verify this, alongside comparisons with non-mutated EGFR cells. 

Interestingly, downregulating HDGF with monoclonal antibodies impaired lung cancer growth and vascularity [155]. Likewise, Shih and colleagues [67] found that miR-214 targeted HDGF in HCC, so miR-214 confers drug resistance in lung cancers but could theoretically target HDGF to hinder tumourigenesis. The balance between the oncogenic and tumour suppressive effects of miR-214 in lung cancer is important to explore. Another factor characterised in lung cancer is Twist1, the previously described upregulator of miR-214 in ovarian cancer [50]. Here, Twist1 again increases miR-214 expression which promotes the transition of epithelial cells into migratory mesenchymal cells, thus stimulating metastasis in lung adenocarcinoma [156].

### 4.7. Breast Cancer

Breast cancer is the leading cause of cancer death in women [125]. In 2018, breast cancer was responsible for 6.6% of all cancer deaths, and nearly one in four cancer diagnoses in women. Inherited BRCA1/2 mutations are prevalent risk factors, as well as hormonal changes from oral contraceptives. Additionally, there is less pressure for early childbearing in developed countries, which increases risk. The high mortality of breast cancer demonstrates an urgent need to find effective treatments. 

Analysis of patient blood serum using TaqMan assays and RT-PCR found that miR-214 could distinguish between benign and malignant breast cancers [40]. Here, miR-214 concentrations were higher in breast cancer patients compared to healthy women, and miR-214 was significantly reduced after surgery. This highlights applications for miR-214 as a diagnostic biomarker to identify breast malignancy. Interestingly, another group observed the downregulation of miR-214 and PTEN in breast cancer tissues [157]. This contradiction is likely due to differences between blood and tumour tissues. Nevertheless, other studies have reported decreased miR-214 expression in breast cancer. Derfoul and colleagues [34] demonstrated an inverse relationship between miR-214 and pro-proliferative Ezh2 expression in a breast cancer cell line. The study noted an in vitro inhibition of proliferation and cell invasion upon expression of miR-214. Targeting of Ezh2 is also consistent with studies on cardiac hypertrophy [37]. Extensive research has shown miR-214 to inhibit breast cancer proliferation and increase apoptosis in vitro by targeting survivin [86] and β-catenin [60], and miR-214 also negatively correlated with the tumour proliferation marker Ki-67 [158]. The latter two studies reported miR-214 downregulation in breast cancer tissues, thus supporting Derfoul and colleagues [34]. However, these groups used the MCF-7 breast cancer cell line, therefore limiting the findings to one environment. Additionally, miR-214 can sensitise breast cancer cells to chemotherapy drugs such as doxorubicin and tamoxifen through directly targeting RFWD2 [79] and UCP2 [88], respectively. Finally, miR-214 targets RNF8, a promoter of metastatic epithelial-mesenchymal transition (EMT) [80]. Importantly, low miR-214 and high RNF8 expression was associated with poor survival in breast cancer patients. In contrast, miR-214-mediated RNF8 targeting encourages ovarian tumourigenesis [81]. Taken together, these studies support miR-214 to suppress breast tumourigenesis through inhibiting proliferation and metastasis, countering chemoresistance, and promoting apoptosis. Further studies should expand on different human cell lines and in vivo models. Screening for further miR-214 targets will also elucidate novel mechanisms.

Despite the extensive research on miR-214 in breast cancer, the field remains polarised. It was reported that high miR-214 expression correlates with poor survival in triple-negative breast cancer (TNBC) patients who lack oestrogen, progesterone and human EGFR receptors [159]. Wang and colleagues observed upregulated miR-214 in breast cancer cell lines alongside increased cell growth and protection against apoptosis through PTEN targeting [35]. This contradicts studies that demonstrated miR-214 downregulation, inhibited proliferation, and increased apoptosis [34,60,86,158]. However, their findings are more representative as miR-214 upregulation was observed in four breast cancer cell lines, and not just MCF-7 [35]. Recently, in vitro and in vivo knockdown of miR-214 with R97/R98 compounds inhibited metastasis and extravasation in TNBC and malignant melanoma [160]. However, this study would have benefited from cell-specific miR-214 knockdown, as systemic silencing may cause unintended effects. Nonetheless, these findings agreed with reports of miR-214 promoting breast cancer cell invasion, partly through targeting p53 [71] or α1-antitrypsin (α1-AT) [57]. As previously described in melanoma, breast cancer cell metastasis was hindered by inhibiting miR-214 or upregulating miR-148b which targets ALCAM and ITGA5 adhesion molecules [38]. Similarly, through targeting the ST6GAL1 enzyme, miR-214 promoted TNBC cell viability, invasion, and the expression of EMT-related proteins like MMPs and N-cadherin [83]. This is contrary to the study by Min and colleagues [80] who proposed that miR-214 inhibited EMT. However, these contradictions may be due to physiological differences between the aggressive TNBC, which makes up 10–20% of cases [83], and other types of breast cancer. Altogether, many studies have shown miR-214 to promote breast cancer metastasis and viability, thus establishing miR-214 inhibition as a possible therapeutic route. Further gain-of-function and loss-of-function studies are needed to clarify the conditions where miR-214 acts as an oncogene or as a tumour suppressor in breast cancer. These inconsistencies may be due to the vast diversity of miR-214 targets and the complex nature of cancer cells. Focusing on a broad range of human breast cancer cell lines and appropriate animal models will help gather insight.

## 5. MicroRNA-214 in Bone Formation

**Figure 6:**
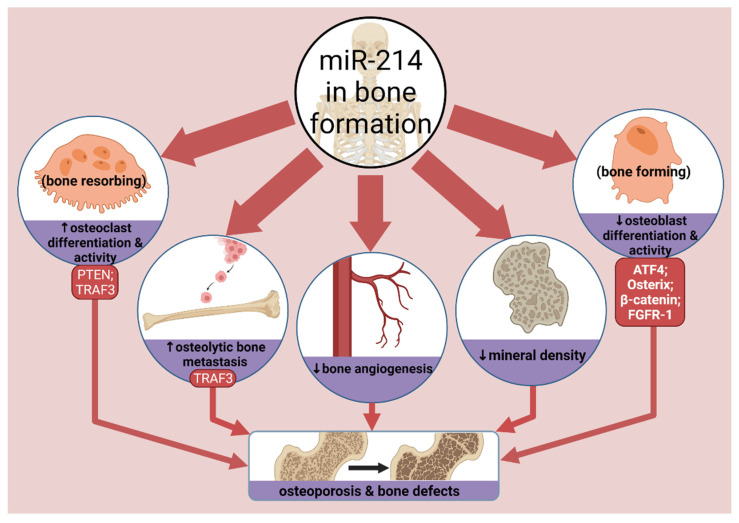
Effects of miR-214 on bone formation. Summary of the detrimental effects of miR-214 on bone formation, with known target mRNA/proteins indicated, as discussed in the main text. Some effects only apply to specific cells or experimental conditions. See the main text of Section 5 for detailed information and references. Created with BioRender.com.

Bone formation, or osteogenesis, is balanced by osteoclasts and osteoblasts [161]. Osteoclasts enable bone resorption, whereas osteoblasts facilitate bone formation. The crosstalk between these two cells, combined with other cytokines and signalling pathways like OPG/RANKL/RANK, ultimately regulate bone homeostasis. Without proper function of these cells, osteoporosis can arise from dysfunctional bone remodelling. The role of miR-214 in regulating bone formation is summarised in Figure 6 and discussed in more detail below.

### 5.1. Osteoblasts

Early microarray analyses found 22 downregulated miRNAs during osteogenesis [162]. However, the scarcity of in vivo studies and the vast number of miRNAs means little is known about their individual function. Nevertheless, miR-125b [163] and miR-2861 [164] can inhibit and promote osteoblast differentiation, respectively. In one of the first studies to characterise a role for miR-214 in osteogenesis, Wang and colleagues detected miR-214 correlating with poor expression of bone formation markers, such as osteocalcin, in humans, [30]. Luciferase reporters identified the pro-osteogenic activating transcription factor 4 (ATF4) as a direct miR-214 target. Similarly, Li and colleagues found exosomal miR-214 to impair osteogenesis in vitro and in vivo [55]. 

Supporting the study of Wang [30], another study found miR-214 to target ATF4 and impair the differentiation of human periodontal ligament stem cells (hPDLSCs) to osteoblasts [58]. This could be hPDLSC-specific, but miR-214 can also inhibit osteogenic differentiation in mesenchymal stem cells (MSC) through targeting FGFR-1 [65], and β-catenin [61]. Additionally, Shi and colleagues found that miR-214 targeted osterix (Osx) [70]; a transcription factor required for osteoblast differentiation. Mice lacking Osx die shortly after birth. The study found that inhibiting miR-214 in the C2C12 myoblast cell line resulted in increased osteogenic markers, including alkaline phosphatase (ALP), Col1α1 and osteocalcin. Interestingly, XBP1 upregulates Osx transcription [165]. In combination with findings that miR-214 targets XBP1 [16], studies should explore the interactions between miR-214 and XBP1-Osx activity. 

Taken altogether, these findings suggest that the therapeutic inhibition of miR-214 can restore bone formation and improve osteoblast activity and differentiation. According to the aptly named Bonewald, Wnt/β-catenin signalling is also implicated in osteocytes [166]. This warrants further study as miR-214 is known to target β-catenin [59,61], so miR-214 may affect Wnt/β-catenin during osteogenesis.

### 5.2. Osteoclasts

Conversely, miR-214 can promote osteoclast differentiation [75]. Osteoclast-specific miR-214 downregulated PTEN and upregulated osteoclast activity and differentiation markers in vivo. In addition, miR-214 promoted excessive osteoclastic resorption in vitro through targeting TRAF3, which encouraged osteolytic bone metastasis (OBM) in breast cancer patients [87]. Antagonising miR-214 could therefore reduce OBM, osteoclastogenesis and bone-resorption, thus encouraging osteoblastic bone formation. Interestingly, miR-214 decreased after exercise and knee-loading in mice, thus increasing mineral density and bone angiogenesis [167,168]. This suggests that exercise prevents osteoporosis through inhibiting miR-214, although this requires further testing.

### 5.3. Therapeutics

Targeting miR-214 with baculovirus gene therapy to treat osteoporosis is promising. A baculovirus vector was engineered to express an miR-214 ‘sponge’ to bind miR-214 and negate its activity [39,169]. This rescued Wnt/β-catenin signalling promoted osteoblast activity and healed osteoporotic defects in ovariectomised (OVX) rats. The lncRNA MALAT1 also sponges miR-214 and can improve osteogenesis by preventing ATF4 downregulation [46]. Similarly, miR-214 sponging was seen with the lncRNA XIST [47], and the pseudogene *PTENP1* [170]. Additionally, delivering cell-specific anti-miR-214 in polyurethane nanomicelle vectors to osteoclasts in OVX mice saw low toxicity and improved bone formation [171]. This is a potential alternative to the dangerous side effects from bisphosphonates used for treating osteoporosis. However, it is important not to overuse anti-miR-214 therapy. Li and colleagues showed that calcific aortic valve disease (CAVD), caused by excessive osteogenesis, can be alleviated by miR-214 which inhibits osteogenic differentiation of valvular interstitial cells [172]. Therefore, miR-214 is protective when moderately antiosteogenic, as it normalises overbearing bone formation.

## 6. MicroRNA-214 in Cell Differentiation

**Figure 7:**
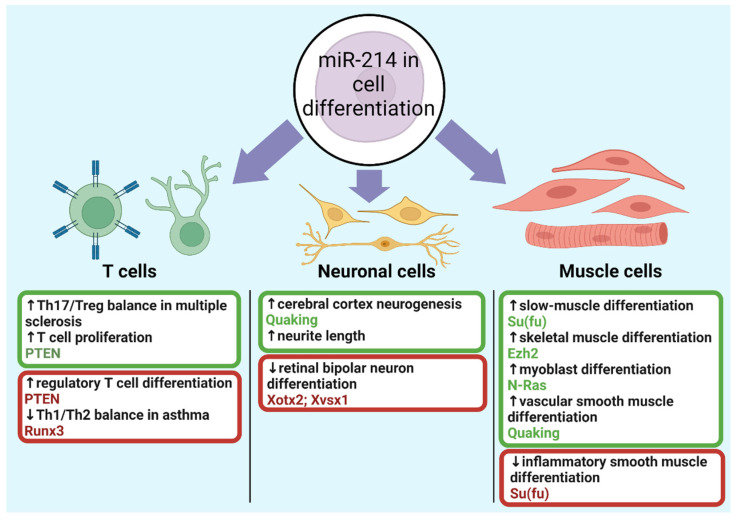
Effects of miR-214 on cell differentiation. Broad summary of the protective (green) and detrimental (red) effects of miR-214 on cell differentiation, with known target mRNA/proteins indicated, as discussed within this review. Some effects only apply to specific cells or experimental conditions. See Section 6 for detailed information and references. Created with BioRender.com.

The effects of miR-214 on the differentiation of muscle cells, neuronal cells and T-cells is summarised in Figure 7 and discussed in detail below.

### 6.1. Muscle Cells

Skeletal muscles develop through myogenesis, where precursors differentiate into myoblasts and eventually myofibres [173]. Myogenesis is controlled through various signalling pathways and transcription factors, although miRNAs are appearing to be increasingly involved. DICER knockouts diminish miRNA processing and cause embryonic lethality in mice due to skeletal muscle hypoplasia [174]. Therefore, understanding miRNAs in myogenesis is important for treating skeletal muscle defects.

Initially, miR-214 was reported to regulate muscle cell development in zebrafish [84]. Here, miR-214 expression was high early in embryonic development, and by targeting Su(fu), hedgehog signalling is activated. Moreover, inhibiting miR-214 reduced differentiation for slow-muscle cell types. Porcine skeletal muscle tissues also revealed differential miR-214 expression between foetal and adult samples [175]. Furthermore, disruption of the *Dnm3os* gene, which encodes the miR-214/miR-199a cluster, caused skeletal defects and postnatal death in mice [49]. These findings suggest a critical role for miR-214 in myogenesis and cell maturation.

Ezh2, the catalytic subunit of the polycomb repressive complex 2 (PRC2), establishes a feedback loop with miR-214 during skeletal muscle cell (SMC) differentiation [31]. PcG proteins suppress miR-214 transcription, but these proteins are released during SMC differentiation. Subsequently, miR-214 downregulates Ezh2 translation which impairs PcG proteins and facilitates further miR-214 transcription, overall, accelerating SMC differentiation. Additionally, C2C12 myoblast differentiation was attenuated upon anti-miR-214 transfection [56]. In agreement, Liu and colleagues described miR-214 to promote C2C12 mouse myoblast differentiation by targeting N-Ras [69]. Altogether, miR-214 can positively regulate muscle cell differentiation. However, these experiments were in vitro and nonhuman specific, so studying in vitro and in vivo human models is needed before considering miR-214 in clinical settings. Contrary to expectations, miR-214 was also downregulated in differentiating C2C12 myoblasts [176]. A plausible explanation is that unsuppressed miR-214 targets promote myogenesis, such as Akt. Future studies should examine the interplay between targets and how their effects outbalance each other. 

Overexpressing miR-214 also promotes embryonic stem cell (ESC) differentiation to vascular smooth muscle cells (VSMCs) both in vitro and in vivo by targeting Quaking [32]. Quaking suppresses the promoter regions of transcription factors involved in VSMC development such as MEF2c, so miR-214 releases their inhibition. This cannot be generalised to all SMC, however, as He and colleagues found miR-214 to inhibit the differentiation to inflammatory SMC by targeting Su(fu) [85]. Additionally, the contrasting effects of miR-214 targets are observed, as miR-214 promotes muscle cell differentiation in zebrafish by also targeting Su(fu) [84]. These differences likely depend on cellular contexts and other transcriptional factors.

### 6.2. Neuronal Cells

Other instances of miR-214 modulating cell differentiation have been realised in neuronal cells. The inhibition of Xotx2 and Xvsx1 mRNAs by miR-214 prevents retinal progenitor cells from differentiating into bipolar neurons in Xenopus [90]. In contrast, another study found miR-214 to be upregulated and increased neurite length in differentiated neuroblastoma cells [177]. Additionally, miR-214 targeted Quaking to promote neurogenesis, the generation of new neurons, during cerebral cortex development [78]. However, miR-214 was also downregulated in differentiated neurons, suggesting an anti-differentiation effect [178]. Surprisingly, inhibiting miR-214 here did not promote neurogenesis, indicating other mechanisms which merit future investigation.

### 6.3. T Cells

Immunomodulatory roles of miR-214 are becoming apparent with T cells. Through targeting PTEN, tumour-secreted miR-214 can promote CD4+ T cell differentiation into regulatory T cells (Tregs) [33]. Tregs inhibit cell-mediated immunity carried out by CD4+ and CD8+ T cells, thus enabling tumours to evade immunity. However, this study showed that anti-miR-214 delivery in microvesicles prevents Treg expansion in vivo, which supports miRNA-based therapy to attenuate tumour growth. Furthermore, CD28 costimulation can upregulate miR-214 to increase T cell proliferation by downregulating PTEN, a negative modulator of T cell signalling [76]. 

Multiple sclerosis (MS) is facilitated by Th17/Treg cell imbalances, which encourage aggressively pathogenic Th17 cells [179]. MiR-214 promotes Treg differentiation here by inhibiting mTOR signalling, therefore balancing Th17/Treg populations. Interestingly, these effects were opposite to miR-27a, which indicates antagonistic interactions with other miRNAs. Similarly, the Runx3 transcription factor can help restore the Th1/Th2 cell balance in asthmatic patients [82]. Runx3 is targeted by five miRNAs, including miR-214. The balance was only restored upon inhibition of all five miRNAs, implying overlapping functions which allow miRNAs to compensate for each other. Overall, studying miR-214 in cell differentiation aids the understanding of regenerative medicine. Viewed alongside osteogenesis studies, it appears miR-214 preferentially promotes the differentiation of specific cells, such as muscle cells [31], and inhibits others, such as osteoblasts [58].

## 7. Conclusions and Future Perspectives

The discovery of miRNAs, in 1993, revolutionised the field of ncRNAs, and studies have since been undertaken to elucidate the regulatory functions of miRNAs in numerous health conditions. This review has described the diverse and complex roles of miR-214 as an oncogene, tumour suppressor, protector against Ca^2+^ overload and oxidative damage, mediator of angiogenesis and pathological fibrosis, and as a regulator in osteogenesis, myogenesis and cellular immunity. Through a vast repertoire of targets, miR-214 influences multiple cellular functions. Oftentimes, contrasting roles are attributed to particular targets. Screening for further miR-214 targets is therefore paramount, as it could reveal a myriad of novel functions in seemingly unrelated diseases. Future studies should also explore external regulators, such as Twist1, as it unlocks possibilities for therapeutic miR-214 modulation. Moreover, miR-214 uptake and secretion are not well understood, which is particularly relevant in tumour microenvironments. Finally, polarised areas remain in the literature, such as in cardiac fibrosis, but more so in breast cancer where contradictions occur even in the same cell lines [34,35]. Further in vivo and in vitro experiments are required to define what dictates miR-214 to act as a tumour suppressor or promoter.

It is important to consider different models, as unique cellular environments yield varying results. In vitro observations may be inconsistent with in vivo animal models, in addition to the disparities between different species. Furthermore, transfecting pre-miR-214 and anti-miR-214 into specific cells is not the same as direct genetic deletions or transgenic animals overexpressing miRNAs systemically. Apart from experimental conditions, opposing interactions between miRNAs and their targets must also be considered, such as miR-148b and miR-199a [38]. Additionally, the less-studied miR-214* requires further investigation. A comprehensive view of miR-214 and the miRNA interactome in human health should be acquired through studying in vitro human cell types, and in vivo cell-specific models using a combination of analytical methods.

Clinical therapies targeting miRNAs are beginning to become a reality. Indeed, a number of pharmaceutical companies have miRNA-based therapeutics in their pipelines with varying success. For example, Roche acquired the miR-122 antagomir, Miravirsen (SPC3649), which is in multiple phase 2 clinical trials for the treatment of hepatitis C virus (HCV) and is a modified locked nucleic acid (LNA) and acts by preventing miR-122 binding to the 5′ UTR HCV genome and ultimately reduces genome transcription [180]. Similarly, Regulus Therapeutics, a company whose pipeline consists purely of miRNA-based therapeutics, has an anti-miRNA oligonucleotide (RG-012) which has been awarded orphan drug status by the FDA for the target of miR-21 to prevent the translation of polysomes in kidney dysfunction for the treatment of patients with Alport syndrome [181]. A third company, miRagen Therapeutics, recently reported the results from a phase 2 clinical trial investigating the safety and tolerability of Remlarsen (MRG-201), a miR-29 miRNA-mimic for the treatment of fibrosis and patients with a history of keloid scars. The specific miR-29 activity being mimicked in this potential treatment is the reduction in collagen expression as well as other proteins involved in fibrosis [182]. These miRNA-based therapeutics utilise the ability to both inhibit and mimic the activity of miRNAs and reflect the important role miRNAs play in the regulation of human disease. It remains to be seen whether therapies aimed specifically at miR-214 will be developed in the future. 

MiR-214 can also act as a diagnostic marker for identifying severe diseases like CAD [41], and this should be explored with other diseases. Many studies also indicate that overexpressing or silencing miR-214 can relieve cardiovascular injury and carcinogenesis both in vitro and in vivo. Recent studies show promise in sponging miR-214 with lncRNAs [46,47] or using cell-specific delivery to relieve osteoporosis [171], although preventing toxicity and ensuring accurate delivery is challenging.

The diversity of miR-214 action is amplified through the variety of targets shown in Table 1, many of which have multiple downstream functions, alongside combinatorial interactions with other targets and miRNAs. Consequently, the therapeutic manipulation of miR-214 must be practised in specific physiological domains to avoid unintended consequences. Care must also be taken to neither negate nor promote the beneficial and detrimental effects of miR-214, respectively. The pleiotropic nature of miR-214 therefore demands a fine balance, for example, in allowing moderate inhibition of osteogenesis to prevent CAVD [172]. Understanding this enigmatic balance through functional studies is a long-term goal for future research in this area.

## Figures and Tables

**Table 1:** MiR-214 targets and associated effects. List of miR-214 targets discussed within this review and associated effects upon miR-214-mediated downregulation of the target mRNA / protein. Individual effects and corresponding references are separated by semicolons.

Target mRNA/Protein	Effect Mediated by miR-214 Downregulation	Reference
α1-AT	Promotes cell viability, invasion, and migration in triple-negative breast cancer.	[57]
ATF4	Inhibits osteoblast activity; inhibits osteoblast differentiation in hPDLSCs.	[30,58]
β-catenin	Reduced activation of pro-proliferative downstream effectors c-myc, TCF-1 and cyclinD1 in hepatocellular carcinoma; inhibits breast cancer proliferation; inhibits osteoblast differentiation.	[59,60,61]
Bcl2l2	Inhibits cervical cancer growth.	[62]
BIM	Inhibits mitochondrial-dependent apoptosis.	[14]
CaMKIIδ	Impairs the regulation of excitation–contraction coupling in the heart.	[14]
CypD	Impairs the opening of the mitochondrial permeability transition pore.	[14]
Ezh1	Inhibits Col1α1 and Col1α3 expression in myofibroblasts.	[19]
Ezh2	Promotes cardiac hypertrophy; inhibits Col1α1 and Col1α3 expression in myofibroblasts; inhibits cervical cancer growth; inhibits breast cancer proliferation and cell invasion; establishes a feedback loop to promote skeletal muscle cell differentiation.	[19,31,34,37,63]
FGFR-1	Inhibits cell invasion in hepatocellular carcinoma; inhibits osteoblast differentiation.	[64,65]
FOXM1	Inhibits cervical cancer growth, invasion and promotes cisplatin sensitivity.	[66]
HDGF	Impairs angiogenic signalling in hepatocellular carcinoma.	[67]
HMGA1	Inhibits cervical cancer growth and invasion.	[68]
ITGA3	Increases melanoma cell migration.	[24]
JNK1	Inhibits proliferation and metastasis in cervical cancer and affects EGFR signaling.	[26]
MEK3	Inhibits cervical cancer progression.	[26]
Mitofusin2 (Mfn2)	Promotes ERK1/2-MAPK activation, cardiac fibroblast proliferation and collagen synthesis.	[17]
NCX1	Attenuates calcium ion overload in the heart.	[14]
NLRC5	Inhibits cardiac fibroblast activation and fibroblast to myofibroblast transition.	[21]
N-Ras	Promotes myogenic differentiation.	[69]
Osterix	Inhibits osteoblast differentiation.	[70]
p53	Promotes breast cancer cell invasion.	[71]
Plexin-B1	Inhibits cervical cancer growth and invasion.	[72]
PTEN	Inhibits PTEN signalling and promotes the PI3K/Akt pathway. Protects against H_2_O_2_-mediated apoptosis in cardiomyocytes; promotes gastric cancer progression; promotes peritoneal metastasis in gastric cancer; promotes ovarian cancer chemoresistance to cisplatin; promotes lung cancer resistance to gefitinib; promotes glycolysis in NSCLC; promotes cell growth and protects against apoptosis in breast cancer; promotes osteoclast differentiation; promotes regulatory T cell differentiation; increases T cell proliferation.	[15,22,23,25,33,35,73,74,75,76]
Quaking	Impairs angiogenic signalling; promotes vascular smooth muscle cell differentiation; promotes neurogenesis during cerebral cortex development.	[32,77,78]
RFWD2	Promotes apoptosis and sensitises breast cancer to doxorubicin.	[79]
RNF8	Inhibits metastatic epithelial–mesenchymal transition in breast cancer; encourages chromosomal instability in ovarian cancer.	[80,81]
Runx3	Impairs the Th1/Th2 cell balance in asthmatic patients when simultaneously targeted by miR-214, miR-371, miR-138, miR-544, and miR-145.	[82]
ST6GAL1	Promotes cell viability, invasion, migration, and epithelial–mesenchymal transition in triple-negative breast cancer.	[83]
Su(fu)	Activates hedgehog signalling and promotes muscle cell differentiation in zebrafish; inhibits inflammatory smooth muscle cell differentiation.	[84,85]
Survivin	Inhibits breast cancer proliferation and increases apoptosis.	[86]
TFAP2C	Increases melanoma cell migration and metastasis, increases progrowth VEGFA and suppresses ERBB2, and regulates many more factors.	[24]
TRAF3	Promotes osteoclast activity and osteolytic bone metastasis in breast cancer patients.	[87]
UCP2	Sensitises breast cancer cells to tamoxifen and fulvestrant.	[88]
XBP1	Impairs angiogenic signalling; impairs hepatocellular carcinoma survival.	[16,89]
Xotx2	Inhibits retinal bipolar neuron differentiation.	[90]
Xvsx1	Inhibits retinal bipolar neuron differentiation.	[90]

## Data Availability

Not applicable.
